# Evaluation of the efficacy of perioperative tranexamic acid in patients with pelvic and acetabular fractures: A systematic review and meta-analysis

**DOI:** 10.1097/MD.0000000000039703

**Published:** 2024-09-20

**Authors:** Yijie Yin, Jiabao Jiang, Chang Zou, Shenbo Huang, Shuai He, Guy Romeo Kenmegne, You Yu, Yue Fang

**Affiliations:** a Department of Orthopedics Surgery, Orthopedic Research Institute, West China Hospital of Sichuan University, Chengdu, China; b Trauma Center, West China Hospital of Sichuan University, Chengdu, China.

**Keywords:** acetabular fractures, pelvic fractures, perioperative management, tranexamic acid

## Abstract

**Background:**

Tranexamic acid (TXA) is commonly used to reduce perioperative bleeding in various surgeries, including acetabular and pelvic fractures treated with open reduction and internal fixation (ORIF). However, research on TXA’s effectiveness and safety in this context is conflicting. To address this, we conducted a systematic review and meta-analysis on TXA’s efficacy and safety in patients with acetabular and pelvic fractures undergoing ORIF.

**Methods:**

We systematically searched Cochrane, PubMed, and EMBASE databases until August 30, 2023. Our evaluation of TXA focused on 6 domains: estimated blood loss (EBL), blood transfusion units, transfusion rates, thromboembolic events, other complications, and surgery duration. Data from these studies were analyzed using RevMan Manager 5.4.

**Results:**

This study included 4 randomized controlled trials with 179 patients with acetabular and pelvic fractures treated with TXA. The analysis showed that TXA did not significantly reduce EBL, packed red blood cell transfusion units, blood transfusion rates, or surgery duration. There was no significant difference in thromboembolic events or other postoperative complications, like surgical wound issues, pneumonia, heterotopic ossification, and sciatic nerve injuries, between the TXA and control groups.

**Conclusion:**

TXA did not demonstrate a significant benefit in reducing perioperative bleeding or complications in patients treated with ORIF for acetabular and pelvic fractures. The utilization of TXA in such clinical scenarios remains a topic necessitating further rigorous investigation to delineate its role in this clinical setting.

## 1. Introduction

Fractures of the acetabulum and pelvis are serious injuries caused by major trauma such as high-impact trauma or car accidents. These fractures can lead to significant bleeding, not only from the fractured ends but also from nearby blood vessels or organs. Hypovolemic shock is reported to be one of the leading causes of death in patients with pelvic and acetabular fractures trauma.^[1]^

Patients with pelvic and acetabular fractures frequently require internal fixation surgery, where blood loss and hypovolemic shock stand out as among the most critical complications. Tranexamic acid (TXA) is a synthetic antifibrinolytic agent that competitively inhibits the lysine-binding sites on plasminogen and tissue plasminogen activator, thereby delaying the dissolution of blood clots.^[2]^ In addition, platelet count and other parameters related to coagulation have been shown to be unaffected by this process.^[3]^ Several orthopedic studies have recommended the use of TXA in major orthopedic surgery, including joint replacement or hip fracture surgery, to safely reduce the amount of blood loss.^[4]^ In a meta-analysis conducted by Yao et al, it was demonstrated that TXA effectively reduced blood loss across various types of surgeries.^[5]^ Recently, 2 trials have been published on the safety and effectiveness of TXA in patients with acetabular and pelvic fractures.^[6,7]^ Considering this is important in the field of traumatic orthopedics, we conducted a systematic review and meta-analysis to elucidate the efficacy of TXA in pelvic and acetabular fractures requiring open reduction and internal fixation (ORIF). Our objective was to investigate the effects of perioperative TXA on blood loss, mean red blood cell transfusion, blood transfusion rate (BTR), and perioperative complications in patients with acetabular and pelvic fractures.

## 2. Method

### 2.1. Search strategy

We conducted our review in accordance with the preferred reporting items for systematic reviews and meta-analyses (PRISMA) guidelines and registered the protocol in PROSPERO. Two independent researchers systematically searched the Medline, Embase, Cochrane databases up until August 30, 2023. The specific search terms included combinations of “pelvis,” “pelvic fractures,” “acetabulum,” “acetabular fracture,” and “tranexamic acid” using Boolean operators to ensure a comprehensive search(see Supplemental File S1, Supplemental Digital Content, http://links.lww.com/MD/N575, which illustrates the search strategy for each database). To avoid overlooking any significant studies, we also manually reviewed the reference lists of pertinent randomized trials, review articles, and previously published meta-analyses, and contacted experts in the field for any unpublished studies to minimize publication bias. Additionally, to resolve discrepancies between the 2 independent reviewers during study selection and data extraction, we implemented a 2-step process. Initially, discrepancies were discussed between the 2 reviewers to reach a consensus. If a consensus could not be reached, a third-party arbitrator with expertise in systematic reviews was consulted. Specifically, the domains evaluated for each study included random sequence generation, allocation concealment, blinding of participants and personnel, blinding of outcome assessment, incomplete outcome data, selective reporting, and other potential biases. Each domain was rated as “low risk,” “unclear risk,” or “high risk” based on predefined criteria. No studies were excluded based on the risk of bias assessment alone; however, the risk levels were taken into account during the interpretation of the results.

### 2.2. Inclusion criteria

In accordance with the PICOs framework, our inclusion criteria were patients diagnosed with pelvic or acetabular fractures who had undergone ORIF surgery. The intervention group received TXA via intravenous infusion, and the control group was administered a placebo with comparable dosage and duration. We focused on predetermined outcomes to evaluate the safety and efficacy of TXA.

Only randomized controlled trials (RCTs) following the Declaration of Helsinki and approved by appropriate ethics committees were included, with a language restriction to English but no geographical limitations. Exclusion criteria comprised the absence of full-text articles, letters, meeting abstracts, and case reports.

The selection process involved 2 independent reviewers who screened titles and abstracts based on predefined inclusion and exclusion criteria. The reviewers then compared their selections, and any discrepancies were identified. Discrepancies were resolved through detailed discussions between the reviewers, guided by the inclusion and exclusion criteria. If consensus could not be reached, a third-party arbitrator with expertise in systematic reviews was consulted. Regular consensus meetings were organized to facilitate discussions on discrepancies. Incomplete data necessitated contacting corresponding authors for further details. Additional citations were sought through manual searches of reference lists in relevant reviews and meta-analyses. Discrepancies were resolved through consensus.

### 2.3. Data extraction

Two researchers individually carried out data extraction from articles identified as eligible through title and abstract review, moving to a full-text assessment of relevant articles. Discrepancies were resolved through discussion and, if necessary, a third author was consulted. These researchers extracted crucial details from each study using a standardized data extraction form, including publication year, study population, participant number, comparators, treatment dosage, blood transfusion triggers, quantity of transfused red blood cell units, number of transfusion recipients, specific adverse events, and data for evaluating the risk of bias in the studies. If necessary, the corresponding authors of the original studies were contacted for clarification.

### 2.4. Quality assessment

Two reviewers independently assessed the methodological quality of the trials included in this study, following the guidelines of the Cochrane Collaboration for Systematic Reviews.^[8]^ The following domains were evaluated:

1. Random sequence generation: rated as “low risk” if adequate random sequence generation methods were described, “high risk” if inadequate methods were used, and “unclear risk” if the method was not described.
2. Allocation concealment: rated as “low risk” if adequate concealment methods were described, “high risk” if concealment was inadequate, and “unclear risk” if not described.
3. Blinding of participants and personnel: rated as “low risk” if blinding was adequately described and maintained, “high risk” if blinding was not used or inadequate, and “unclear risk” if not described.
4. Blinding of outcome assessment: rated as “low risk” if outcome assessors were adequately blinded, “high risk” if blinding was inadequate, and “unclear risk” if not described.
5. Incomplete outcome data: rated as “low risk” if all participants were accounted for and missing data handled appropriately, “high risk” if there were high dropout rates or inadequate handling of missing data, and “unclear risk” if not described.
6. Selective reporting: rated as “low risk” if all prespecified outcomes were reported, “high risk” if some outcomes were selectively reported, and “unclear risk” if it was not clear whether outcomes were selectively reported.
7. Other bias: rated as “low risk” if no other biases were identified, “high risk” if other potential biases were present, and “unclear risk” if the risk of other biases was not clear.

Each domain was assessed independently by 2 reviewers. Discrepancies in assessments were resolved through discussion or consultation with a third reviewer.

### 2.5. Statistical analysis

Statistical analysis was executed independently by 2 reviewers utilizing RevMan software (Version 5.4; The Nordic Cochrane Centre, The Cochrane Collaboration, 2020). Continuous variables such as estimated blood loss (EBL) were assessed through the mean difference (MD) with a 95% confidence interval (95% CI) between the TXA and control groups, while dichotomous outcomes like BTR were evaluated using the risk ratio with a 95% CI. We assessed heterogeneity using the I² statistic, which describes the percentage of variation across studies due to heterogeneity rather than chance. Fixed-effects model assumes that all included studies estimate the same underlying effect size. This model is appropriate when heterogeneity is low, indicating that the differences between study outcomes are likely due to random variation rather than true differences in effect sizes. Random-effects model assumes that the true effect size varies between studies due to real differences in study populations, interventions, or other factors. This model is more appropriate when there is substantial heterogeneity, as it accounts for the variability both within and between studies. For outcomes with *I*² ≤ 50%, indicating low heterogeneity, we used a fixed-effects model. For outcomes with *I*² > 50%, indicating substantial heterogeneity, a random-effects model was applied. This approach ensured that our meta-analysis appropriately accounted for variability in study outcomes. Sensitivity analyses were conducted to assess the robustness of our findings. The main purpose of sensitivity analysis in a meta-analysis is to assess the robustness and reliability of the overall findings. By systematically excluding individual studies, we can determine the influence of each study on the pooled effect size and identify any potential outliers or studies that disproportionately affect the results. We conducted a sensitivity analysis by sequentially excluding each study from the meta-analysis. This involved re-running the meta-analysis multiple times, each time leaving out 1 study, to observe changes in the pooled effect size, confidence intervals, and heterogeneity (I² statistic). For each exclusion, we documented the following: change in effect size: any significant changes in the pooled effect size were noted, confidence interval shifts: we observed any notable widening or narrowing of the confidence intervals, heterogeneity adjustment: we recorded changes in the I² statistic to see if excluding certain studies reduced overall heterogeneity, consistency of findings: we assessed whether the overall conclusions remained consistent despite the exclusion of individual studies. We sequentially excluded each study from the meta-analysis to observe changes in pooled effect sizes, confidence intervals, and heterogeneity. This process helped identify any studies that disproportionately influenced the results. The results, graphically illustrated through forest plots, were considered statistically significant at *P* < .05. Although a funnel plot was employed to assess publication bias, the interpretation was constrained due to the limited number of included studies, restricting the analysis to partial outcomes.

## 3. Result

### 3.1. Literature search

**Figure 1.**
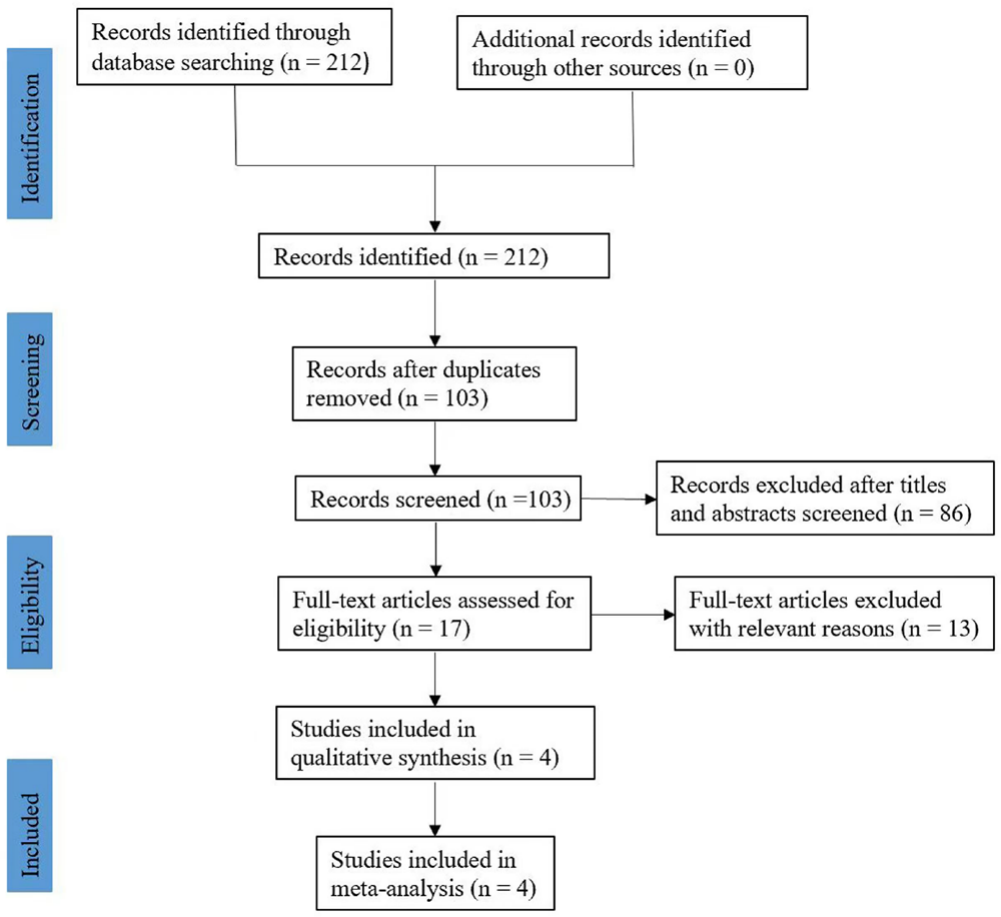
Flow chart of search and inclusion results.

We reviewed 52 abstracts, 21 of which qualified for full-text assessment. Ultimately, 4 trials encompassing 352 participants met the criteria for inclusion in the meta-analysis (see Fig. 1, which illustrates the Flow chart of search and inclusion results).

### 3.2. Description of included study

Table 1 presents the characteristics of the included trials. The studies were published in 2017, 2019, and 2 in 2022, with population sizes ranging from 63 to 108. All trials administered TXA intravenously. Refer to Table 2 for detailed information.

**Table 1:** The main characteristics of the included studies.

Study(year)	Country	No. of patients		Female		Age		Preoperative Hb (g/L)		Thromboprophylaxis	Thrombus screen	Outcome measures	Follow-up (month)
				T	C	T	C	T	C				
Lack et al 2017^[9]^	America	42	46	11	12	41.7	39.7	113	115	LMWH, intermittent compression devices	Clinical	BTR, BTU, EBL, surgical time, surgery length, complications, cell saver cases	NR
Mohamed et al 2022^[6]^	Egypt	54	54	15	17	32.4	37.9	107	101	LMWH, compression stockings	Clinical	TBL, THL, IBT, BTU, complications, Hb and Hct at surgery and day 1 and day 3,surgery length	26
Sen et al 2022^[7]^	India	36	27	6	6	49.1	44.3	118	114	LMWH	Color doppler ultrasonography, and CTA	EBL, drain output, BTR, BTU, Hb and Hct on day 2, surgery length, complications	NR
Spitler et al 2019^[10]^	America	47	46	11	14	47.2	42.8	NR	NR	LMWH and mechanical sequential compression devices	duplex ultrasound	Preoperative Hct, IBT, cell saver cases, TBL, EBL, surgery length, postop Hct on day 1, day 2 and day 3, IBTR, TBTR, complications	NR

BTR = blood transfusion rate, BTU = blood transfusion unit, C = control group, CTA = computerized tomography Angiography, Hb = hemoglobin, Hct = hematocrit, IBT = intraoperative blood transfusion, IBTR = intraoperative blood transfusion rate, LMWH = low molecular weight heparin, NR = not reported, T = tranexamic acid (TXA) group, TBRT = total blood transfusion rate, THL = total hemoglobin loss.

**Table 2:** The surgical information of included studies.

Study (year)	Fracture	Surgical procedure	intervention	Control	Transfusion trigger
Lack et al 2017^[9]^	Acetabular fracture	ORIF	IV 10 mg/kg TXA within 30 minutes before surgery, and the same dose over a 4-hour period during surgery	Equal volume and rate of normal saline	Hb < 70 g/L; patients with acute blood loss anemia
Mohamed et al 2022^[6]^	Pelvic and actabular fracture	ORIF	IV 15 mg/kg TXA after induction of anesthesia, and the same dose again 3 hours later	Placebo	Hb < 80 g/L or Hb < 90 g/L for cardiac patients
Sen et al 2022^[7]^	Acetabular fracture	ORIF	IV 10 mg/kg TXA 15 minutes prior to incision, and the same dose again 3 hours later	The same volume of normal saline	Hb < 80 g/L
Spitler et al 2019^[10]^	Pelvic, acetabular and proximal femoral fracture	ORIF	IV 15 mg/kg TXA just before incision, and the same dose again 3 hours later	Without TXA	Hb < 80 g/L or Hb < 90 g/L for patients with significant cardiac or pulmonary disease; all patients who demonstrated symptomatic anemia postoperatively

Hb = hemoglobin, IV = intravenous injection, ORIF = open reduction and internal fixation, TXA = tranexamic acid.

### 3.3. Quality assessment of individual trials

**Figure 2.**
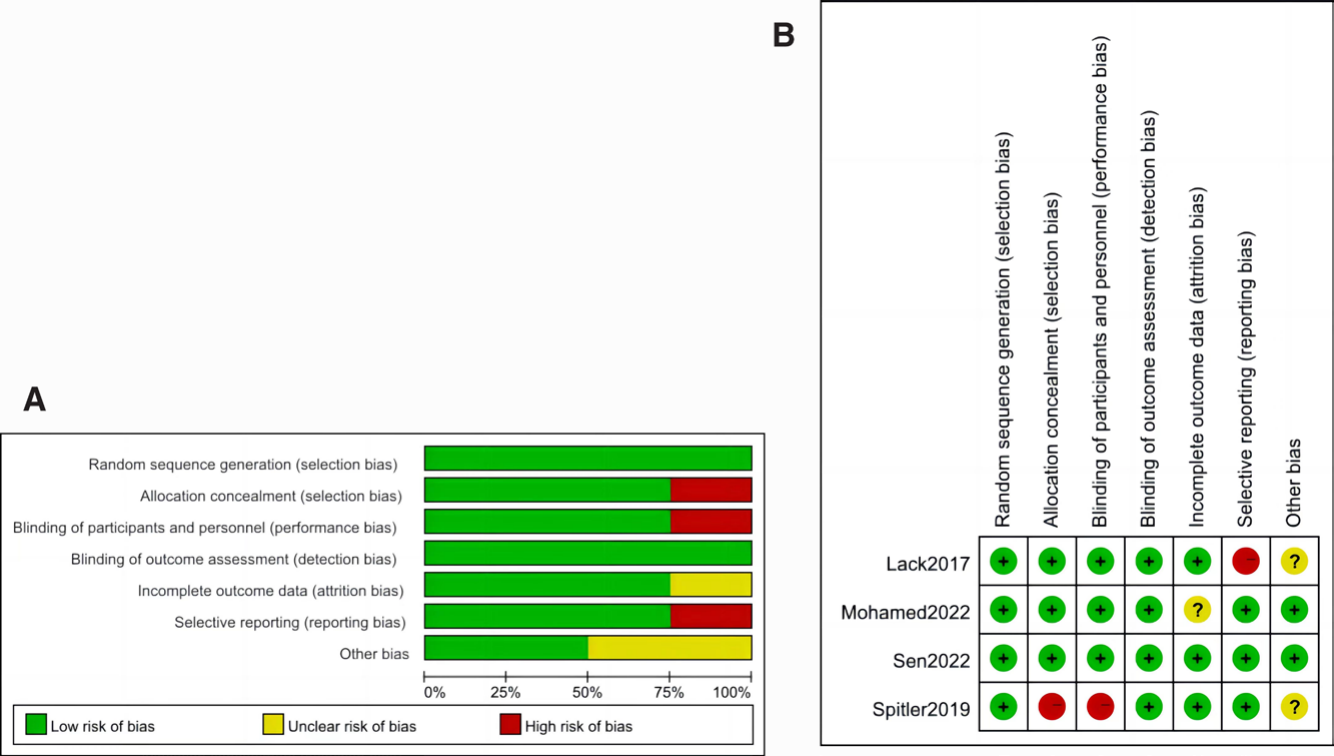
(A) Risk of bias summary: low-risk of bias in green; unclear risk of bias in yellow; high risk of bias in red. (B) Bias risk graph: each item of bias risk is depicted as a percentage, representing the distribution of various bias risk levels for each item across all studies included.

All 4 studies in our review employed adequate random sequence generation.^[6,7,9,10]^ Three of them ensured allocation concealment,^[6,7,9]^ while the last one did not report on this aspect, leading to high risk.^[10]^ Blinding of participants and personnel was maintained in 3 studies,^[6,7,9]^ but was high risk in the remaining one due to a lack of reporting.^[10]^ Though blinding of outcome assessment was not documented in any of the studies, we assessed them as low-risk given the unlikelihood of the outcomes being influenced by lack of blinding. All studies presented complete outcome reports and data, and no evident sources of bias were identified across the trials. Figure 2A and B respectively depict the risk of bias summary and graph of the analyzed studies.

### 3.4. Meta-analysis of estimated blood loss

**Figure 3.**
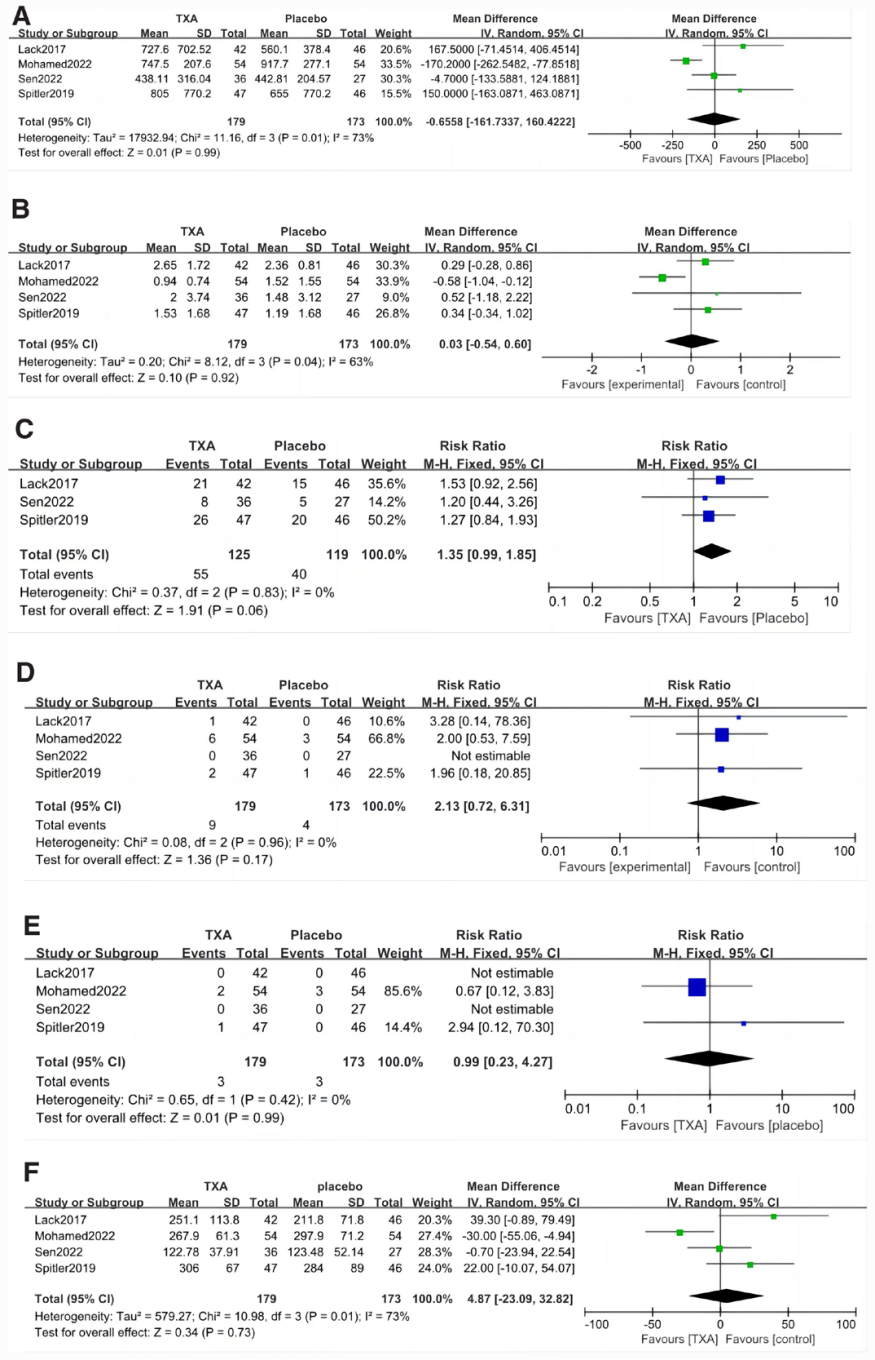
(A) Forest plot showing estimated blood loss of TXA group versus control group. (B) Forest plot showing blood transfusion units of TXA group versus control group. (C) Forest plot showing blood transfusion rate of TXA group versus control group. (D) Forest plot showing thromboembolic events of TXA group versus control group. (E) Forest plot showing other complications of TXA group versus control group. (F) Forest plot showing surgery length of TXA group versus control group.

All studies reported EBL, which included 179 and 173 patients in the TXA and control groups, respectively. We found that perioperative administration of TXA did not reduce blood loss in these patients (MD = −0.66; 95% CI: −161.7 to − 160.4; *P* = .01) [see Fig. 3A, which illustrates the EBL of included study].

### 3.5. Meta-analysis of blood transfusion units

Three studies reported the number of units of TXA transfused in patients in the TXA and control groups, and 1 study reported the means of blood transfusion (The single transfusion blood unit was considered to add 55 g to the Hb level). We found that perioperative administration of TXA did not reduce packed red blood cell transfusion units in patients with pelvic and acetabular fractures (MD = 0.03; 95% CI: −0.54 to 0.60; *P* = .04) [see Fig. 3B, which illustrates the forest plot of blood transfusion units of TXA group vs control group].

### 3.6. Meta-analysis of blood transfusion rate

All studies reported the number of patients received blood transfused, which included fifty-five and forty patients in the TXA and control groups, respectively. The results of pooled analyses are shown in Figure 3C. The results showed that the transfusion rate of the TXA group has no significant difference with the control group. (RR = 1.35; 95% CI: 0.99 to 1.85; *P* = .83).

### 3.7. Meta-analysis of thromboembolic events

Thromboembolic events were reported in 4 studies, encompassing incidents of lower extremity deep-vein thrombosis (DVT) and pulmonary embolism (PE). In the TXA and control groups, there were 4 and 2 cases of PE and 5 and 2 cases of DVT, respectively. Pooled analyses displayed in Figure 3D reveal no significant difference between the groups regarding the incidence of thromboembolic events (RR = 2.13; 95% CI: 0.72 to 6.31; *P* = .96).

### 3.8. Meta-analysis of other complications

All 4 studies reported complications after surgery, which included 3 and 3 patients in the TXA and control groups, respectively. Complications included surgical wound complications, pneumonia, heterotopic ossification, and sciatic nerve injury. The results of pooled analyses are shown in Figure 3E. The results showed that the incidence of complications in the TXA group has no significant difference with the control group (RR = 0.99; 95% CI; 0.23 to 4.27; *P* = .42).

### 3.9. Meta-analysis of length of surgery

The surgery duration was documented across all 4 studies, revealing no significant difference between the TXA and control groups (MD = 4.87; 95% CI: −23.09 to 32.82; *P* = .01) [see Fig. 3F, which illustrates the forest plot showing surgery length of TXA group vs control group].

### 3.10. Sensitivity analysis

We conducted a sensitivity analysis by sequentially excluding each study to observe any alterations in the consolidated outcomes. It was observed that the exclusion of the trial by Mohamed et al, 2022, led to a decrease in I² from 63% to 0% for transfusion units and from 73% to 1% for EBL. Despite these variations in I², no notable heterogeneity was identified in other outcome measures. Furthermore, the omission of any single study did not materially impact the overarching conclusion that TXA does not significantly affect EBL, transfusion units, transfusion rate, thrombosis event rate, other complication rates, and surgery duration.

### 3.11. Publication bias

These results held true under both random-effects and fixed-effects statistical models. Additionally, no signs of publication bias were discerned, whether assessed through funnel plot visualization or statistical evaluation.

## 4. Discussion

In this research, we carried out a systematic review and meta-analysis to scrutinize the effectiveness and safety of tranexamic acid in patients experiencing pelvic and acetabular fractures who underwent ORIF. Our findings indicate that the use of TXA in this cohort does not diminish EBL, the number of transfusion units, or the transfusion rate. Additionally, it does not confer any benefit in reducing the surgery duration or the BTR when compared to the control group. Encouragingly, it does not elevate the risk of thromboembolic events or other postoperative complications such as infections at the wound site, pneumonia, heterotopic ossification, and sciatic nerve injuries.

Acetabular and pelvic fractures represent serious traumatic injuries, which are commonly caused by high-energy traumas, including car crashes and falls from considerable heights.^[11]^ These injuries can have grave repercussions including death and permanent disability, posing substantial burdens on societies and families. Although treatment options vary, encompassing conservative methods and less invasive surgical interventions, ORIF remains the preferred choice, especially for cases unsuitable for percutaneous fixation. ORIF provides optimal visualization and promises better long-term outcomes for individuals with both acetabular and pelvic ring fractures.^[12]^

However, despite the increasing sophistication of surgical techniques for fracture treatment, hemorrhage due to severe pelvic trauma remains a serious concern for clinicians. The heightened morbidity linked with pelvic injuries arises from factors such as hemorrhage, extended operative durations, thromboembolic events, and concurrent traumatic injuries.^[13–16]^ Moreover, research indicates that the volume of bleeding plays a pivotal role in determining the patient’s prognosis.^[15]^ In the case of hemodynamically unstable patients, immediate emergency intervention takes precedence, involving assertive management of critical comorbid injuries following the ABCDEF rescue protocol devised by McMurtry. This protocol delineates focus areas as airway, bleeding, central nervous system, digestion, excretion, and fracture management, sequentially. Hemorrhage control strategies involve methods such as allogeneic blood transfusion, employing pelvic banding to reduce pelvic volume, and utilizing pelvic tamponade.^[17]^ However, it should be noted that allogeneic transfusions come with associated risks including the potential transmission of infectious diseases, adverse transfusion reactions, acute lung injury, DVT, and susceptibility to postoperative infections.^[18,19]^ Moreover, in numerous countries and regions globally, securing a sufficient supply of blood products is frequently challenging, underscoring the significance of minimizing allogeneic transfusions. Therefore, implementing strategies to reduce blood loss is crucial in decreasing morbidity and social burden.^[20,21]^ Given these risks, there is a growing reliance on hemostatic agents like tranexamic acid in the perioperative management of orthopedic cases.^[20,22]^

In recent times, the utilization of TXA has dramatically increased, marking it as a powerful tool in perioperative blood management. TXA functions by impeding fibrinolysis, which it accomplishes by obstructing the lysine-binding sites of plasminogen connected to fibrin, thereby dissociating plasminogen from the fibrin surface.^[23]^ Its application has permeated a variety of surgical fields, including cardiothoracic surgery, neurosurgery, and obstetrics, demonstrating its extensive applicability in contemporary surgical practices.^[24]^ In orthopedic surgery, TXA has demonstrated substantial effectiveness in reducing blood loss during various procedures including spinal fusion, joint replacements, and hip fracture surgeries.^[25,26]^ However, while it showcases encouraging outcomes, the efficacy and safety of TXA in surgeries pertaining to pelvic and acetabular fractures remain a subject necessitating deeper investigation. The best administration route of TXA during the perioperative period in orthopedic surgery is currently controversial. It can be administered intravenously, orally, through nasal modes, or locally.^[27,28]^ A consensus on the optimal administration route has yet to be reached.^[29]^ The documented techniques for topically applying TXA in hip fractures encompass the injection of TXA beneath the soft tissue of the fascia surrounding the fracture or the local intramuscular injection of TXA.^[28,30]^ Intra-articular and periarticular tissue injections of TXA have also been documented in the context of joint replacement for femoral neck fractures.^[31]^ Some studies suggest that the advantages of local administration include achieving the maximum concentration at the bleeding site and minimal systemic absorption, thereby reducing the risk of thromboembolic complications and other potential risks.^[32]^ However, other research argues that local administration does not significantly differ in reducing postoperative blood loss and transfusion rates in intertrochanteric fractures.^[28]^ It is noteworthy that studies generally agree that TXA does not increase the risk of thrombotic events, but it is still advised not to be used in conjunction with coagulants. Caution is recommended when administering tranexamic acid to individuals with preexisting thrombotic disorders or consumptive coagulopathy to avoid an increased risk of thromboembolism. A research indicates that TXA exhibits a dose-dependent toxic effect on cartilage, tendon, and synovial tissue, and underscores the necessity for caution when considering intra-articular administration of TXA.^[33]^ There is a need for further human clinical trials in order to clarify the long-term safety of TXA topical application. Currently, there is no reported evidence regarding the anti-inflammatory or anti-edematous effects of TXA.

In our investigation, we determined that the perioperative administration of TXA did not significantly reduce the EBL in patients subjected to acetabular or pelvic fracture surgeries. Even though TXA has a proven track record in minimizing blood loss in joint and spinal surgeries,^[34–37]^ its effectiveness remained unclear in the context of our study. The preliminary analysis displayed a high heterogeneity of 73%, suggesting considerable inconsistency and potentially questioning the trustworthiness of our conclusions. However, upon excluding an outlier study by Mohamed et al (2022), the heterogeneity drastically decreased to 1%. Despite this adjustment, the TXA cohort did not showcase any statistically significant benefit over the placebo group in terms of reducing EBL or decreasing the quantity of transfusion units needed. In addition, a clinical trial showed that TXA reduced blood loss and transfusion rates in elderly patients with femoral neck fracture.^[31]^ We theorize that the lack of observed beneficial effects of TXA could be due to the distinct nature of pelvic and acetabular fractures. A recent review has addressed similar issues, suggesting that patients with acetabular fractures combined with pelvic ring fractures often have multiple injuries and vary in fracture severity, and that the efficacy of TXA may simply be limited by the complexity of these patients.^[38]^ TXA exerts its influence on the entire coagulation process by inhibiting the breakdown of blood clots.^[23]^ This mechanism allows TXA to reduce bleeding originating from soft tissue wounds or blood vessels, facilitating a more controlled and effective hemostasis during orthopedic surgery. The exposed cancellous bone surfaces in acetabular fractures serve as the primary source of bleeding, and this form of bleeding remains unaffected by the administration of these antifibrinolytic agents.^[9]^ Primarily, the intraoperative bleeding stems from fractured bone ends, where TXA has not historically shown efficacy in curtailing bone bleeding volume.^[39]^ Moreover, the efficacy of TXA might be obscured by numerous confounding variables, such as differing preoperative hemoglobin concentrations, various fracture shapes, disparate waiting periods before surgery, and the range of existing comorbidities among patients. Notably, past research has pointed to the association of low preoperative hemoglobin levels, intricate fracture patterns, and longer surgery durations with increased transfusion rates.^[9]^Therefore, although TXA did not outperform the placebo in decreasing EBL and transfusion necessities in this study, it remains imperative to delve deeper into the potential influences of various confounding aspects. Further studies should be undertaken to definitively discern the role of TXA in managing patients with acetabular and pelvic fractures.

Theoretically, TXA use could heighten the incidence of thromboembolic events. In a comprehensive clinical trial focused on gastrointestinal bleeding, it was observed that individuals who were administered TXA exhibited a heightened risk for experiencing DVT compared to participants in the control group.^[40]^ However, our study did not observe an elevated risk of complications such as DVT, aligning with previous research.^[25,41,42]^ Contrary to some meta-analyses that reported a decreased rate of wound complications associated with TXA in treating other fractures,^[43–46]^ our study did not find a reduction in wound infection risks with TXA usage. This inconsistency might be attributed to a low incidence of cases, with only 1 study^[16]^ reporting a single superficial infection occurrence in the TXA group out of the 4 investigations incorporated in our research. Furthermore, our study identified no increased risk of complications including pneumonia, ectopic ossification, and sciatic nerve injury when using TXA. We also found no significant discrepancy in surgery duration between the TXA and control groups.

In all the clinical trials encompassed in our analysis, TXA was administered via the intravenous route. Despite this prevalent approach, the optimal route of administration for orthopedic surgeries remains unclear.^[29]^ Utilizing TXA topically could potentially offer several benefits over intravenous administration, including delivering a concentrated amount directly at the hemorrhage sites and minimizing systemic absorption, which might consequently reduce the likelihood of thromboembolic events and other adverse complications.^[32]^ While there have been investigations into the local application of TXA in patients suffering from hip fractures,^[28]^ there is a conspicuous absence of research addressing its topical application in the context of individuals enduring pelvic and acetabular fractures. As we move forward, it is imperative for future clinical studies to prioritize determining the most efficacious route for administering TXA.

Our research is accompanied by several limitations. Firstly, while we focused solely on RCTs to reduce the bias typically found in retrospective studies, this approach limited the pool of available studies and participants, leaving a relatively modest number of subjects included, which could have introduced another form of bias. Secondly, due to the limited number of studies accessible, we couldn’t evaluate the potential effects of variations in TXA administration, such as differences in dosage, frequency, and timing, on the outcomes. Thirdly, a deficiency in the long-term functional follow-up data in the studies reviewed restricts the depth of our analysis, as we could not gauge the prolonged effects and recovery rate posttreatment. Furthermore, we could not analyze data pertaining to intraoperative or postoperative blood loss, and the involvement of autologous blood cell transfusion techniques might have altered transfusion metrics, which consequently might affect the derived conclusions on the efficacy of TXA. Lastly, acetabular fracture and pelvic fracture have great differences in fracture morphology, and their effects on blood loss and blood transfusion should not be ignored. The absence of reported proportions of patients with pelvic or acetabular fractures in specific studies could potentially impact the accuracy of the final results.To sum up, initiating prospective randomized controlled clinical studies that entail larger sample sizes becomes an imperative step in substantiating the efficiency of TXA in treating individuals with pelvic and acetabular fractures. However, these patients often present with severe, multifaceted injuries, which renders the organization of RCTs a challenging endeavor. Given these factors, it is crucial to approach the findings of this research with a nuanced perspective, recognizing the potential for future studies to further elucidate the role and efficacy of TXA in this medical context.

Given these limitations, further research is warranted to better understand the role of TXA in pelvic and acetabular fractures. We recommend the following areas for future investigation: larger, multicenter RCTs to confirm the efficacy and safety of TXA in patients with pelvic and acetabular fractures, optimal dosage and administration routes, patient subgroup analysis, long-term follow-up studies. And to overcome the limitations noted in our review, future studies should: standardize study designs, encourage comprehensive reporting, include long-term data.

## 5. Conclusion

This systematic review and meta-analysis showed that intravenous administration of TXA did not serve to reduce EBL, blood transfusion units, or transfusion rates in patients with pelvic and acetabular fractures who underwent ORIF. However, it also does not increase the risk of thromboembolic events nor does it contribute to the risk of wound complications and heterotopic ossification. Thus, there is insufficient evidence to support the routine use of tranexamic acid in the perioperative period of pelvic and acetabular surgery.

Our findings underscore the need for further large-scale, multicenter RCTs to determine the optimal use of TXA in this context. Additionally, future studies should explore the most effective dosage and administration routes, assess the long-term outcomes, and investigate the underlying mechanisms of TXA’s effects. By addressing these gaps, future research can provide clearer guidance on the role of TXA in managing perioperative bleeding in patients with pelvic and acetabular fractures.

## Acknowledgments

We would like to thank the West China Hospital of Sichuan University for allowing us to conduct this research and providing valuable data that was used in this study.

## Author contributions

**Data curation:** Yijie Yin, Shenbo Huang, Shuai He, Guy Romeo Kenmegne.

**Investigation:** Shuai He, Guy Romeo Kenmegne, You Yu.

**Methodology:** Yijie Yin.

**Supervision:** Chang Zou, Yue Fang.

**Validation:** Jiabao Jiang, Chang Zou, Yue Fang.

**Writing – original draft:** Yijie Yin.

**Writing – review & editing:** Yijie Yin, Jiabao Jiang, Shenbo Huang, Guy Romeo Kenmegne, Yue Fang.

## Supplementary Material


