# Supplementary material for: Evaluation of the efficacy of perioperative tranexamic acid in patients with pelvic and acetabular fractures: A systematic review and meta-analysis
Source: Medicine (Baltimore). 2024 Sep 20;103(38):e39703. doi: 10.1097/MD.0000000000039703 (PMC11419542; doi:10.1097/MD.0000000000039703)
Supplement: Supplementary file 1 [file medi-103-e39703-s001.docx]

| Pubmed | ("Pelvis"[MeSH Terms] OR (("pelvics"[All Fields] OR "Pelvis"[MeSH Terms] OR "Pelvis"[All Fields] OR "pelvic"[All Fields]) AND ("fractur"[All Fields] OR "fractural"[All Fields] OR "fracture s"[All Fields] OR "fractures, bone"[MeSH Terms] OR ("fractures"[All Fields] AND "bone"[All Fields]) OR "bone fractures"[All Fields] OR "fracture"[All Fields] OR "fractured"[All Fields] OR "fractures"[All Fields] OR "fracturing"[All Fields])) OR (("pelvics"[All Fields] OR "Pelvis"[MeSH Terms] OR "Pelvis"[All Fields] OR "pelvic"[All Fields]) AND ("fractur"[All Fields] OR "fractural"[All Fields] OR "fracture s"[All Fields] OR "fractures, bone"[MeSH Terms] OR ("fractures"[All Fields] AND "bone"[All Fields]) OR "bone fractures"[All Fields] OR "fracture"[All Fields] OR "fractured"[All Fields] OR "fractures"[All Fields] OR "fracturing"[All Fields])) OR (("pelvi"[All Fields] OR "Pelvis"[MeSH Terms] OR "Pelvis"[All Fields]) AND ("fractur"[All Fields] OR "fractural"[All Fields] OR "fracture s"[All Fields] OR "fractures, bone"[MeSH Terms] OR ("fractures"[All Fields] AND "bone"[All Fields]) OR "bone fractures"[All Fields] OR "fracture"[All Fields] OR "fractured"[All Fields] OR "fractures"[All Fields] OR "fracturing"[All Fields])) OR (("pelvi"[All Fields] OR "Pelvis"[MeSH Terms] OR "Pelvis"[All Fields]) AND ("fractur"[All Fields] OR "fractural"[All Fields] OR "fracture s"[All Fields] OR "fractures, bone"[MeSH Terms] OR ("fractures"[All Fields] AND "bone"[All Fields]) OR "bone fractures"[All Fields] OR "fracture"[All Fields] OR "fractured"[All Fields] OR "fractures"[All Fields] OR "fracturing"[All Fields])) OR "acetabulum"[MeSH Terms] OR ((("acetabular"[All Fields] OR "acetabulare"[All Fields]) AND ("fractur"[All Fields] OR "fractural"[All Fields] OR "fracture s"[All Fields] OR "fractures, bone"[MeSH Terms] OR ("fractures"[All Fields] AND "bone"[All Fields]) OR "bone fractures"[All Fields] OR "fracture"[All Fields] OR "fractured"[All Fields] OR "fractures"[All Fields] OR "fracturing"[All Fields])) OR (("acetabular"[All Fields] OR "acetabulare"[All Fields]) AND ("fracture"[All Fields] OR "fractural"[All Fields] OR "fracture s"[All Fields] OR "fractures, bone"[MeSH Terms] OR ("fractures"[All Fields] AND "bone"[All Fields]) OR "bone fractures"[All Fields] OR "fracture"[All Fields] OR "fractured"[All Fields] OR "fractures"[All Fields] OR "fracturing"[All Fields])))) AND "Tranexamic Acid"[MeSH Terms] |
| --- | --- |
| Cochrane | Pelvis OR "Pelvic Fractures" OR "Pelvic Fracture" OR "Pelvis Fractures" OR "Pelvis Fracture" OR acetabulum OR "acetabular fracture" OR "acetabular fractures" in All Text AND "Tranexamic Acid" in All Text - in Cochrane Reviews, Cochrane Protocols, Trials, Clinical Answers, Editorials, Special Collections (Word variations have been searched) |
| Embase | ((Pelvis) OR (Pelvic Fractures) OR (Pelvic Fracture) OR (Pelvis Fractures) OR (Pelvis Fracture) OR (acetabulum) OR ((acetabular fracture) OR (acetabular fractures)) AND (Tranexamic Acid). {Including Related Terms)  Search terms used:acetabulum， acetabula， acetabulums， bone structure of acetabulum， acetabular acetabulum， pelvic fractures， fracture pelvis， fractures pelvic， pelvic fracture， pelvis fracture， pelvis fractures， pelvic fracture， pelvis fracture， fracture of pelvis， pelvic fractures， fracture pelvis， fractures pelvic， pelvis fractures， pelvic fractures， fracture pelvis， fractures pelvic， pelvic fracture，pelvis fracture，tranexamic acid，trans 4 aminomethyl, cyclohexanecarboxylic acid, acid tranexamic, amca, amcha |
